# GraXRS-Dependent Resistance of Staphylococcus aureus to Human Osteoarthritic Synovial Fluid

**DOI:** 10.1128/mSphere.00143-21

**Published:** 2021-03-10

**Authors:** David W. Watson, Santiago L. Iglesias, Edward M. Vasarhelyi, David E. Heinrichs

**Affiliations:** a Department of Microbiology and Immunology, University of Western Ontario, London, Ontario, Canada; b Division of Orthopaedic Surgery, University of Western Ontario, London Health Sciences Centre, University Hospital, London, Ontario, Canada; c Department of Orthopedic Surgery, Sanatorio Allende, Córdoba, Argentina; University of Rochester

**Keywords:** synovial fluid, joint, infection, iron, GraXRS, *S. aureus*, MRSA, *Staphylococcus aureus*, cationic peptides, osteoarthritis

## Abstract

Osteoarthritis is the most prevalent joint disease in the United States, with many patients requiring surgical replacement of the affected joint. The number of joint arthroplasty procedures performed each year is increasing, and infection is a leading cause of implant failure. Staphylococcus aureus is the most frequently isolated organism associated with periprosthetic joint infections of the knee or hip, and due to the emergence of antibiotic-resistant strains, treatment options are limited. Here, we show that synovial fluid from osteoarthritic patients is iron restrictive toward S. aureus and, for strains representing the clonal lineages USA100, USA200, USA400, and USA600, bactericidal. Remarkably, community-associated methicillin-resistant S. aureus (CA-MRSA) strain USA300-LAC was highly resistant to synovial fluid killing but could be sensitized to killing by mutation of the GraXRS regulatory system and GraXRS-regulated *mprF* gene or by small-molecule inhibition of GraR. Thus, we propose the GraXRS-VraFG regulatory system and *mprF* as targets for future therapeutics for treatment of S. aureus bone and joint infections.

**IMPORTANCE** Osteoarthritis, a degenerative disease that results in the breakdown of joint cartilage and underlying bone, is the most prevalent joint disease in the United States. Surgical intervention, including total joint replacement, is a clinically effective procedure that can help to restore the patient’s quality of life. Unfortunately, joint replacement procedures come with a risk of infection that is estimated to occur in 1 to 2% of cases, and periprosthetic joint infection (PJI) is a leading cause of implant failure, requiring revision surgery. Staphylococcus aureus is well known for its ability to cause PJIs and was found to be the most frequently isolated organism from PJIs of the knee or hip. Antibiotic-resistant strains can often limit treatment options. In this study, we demonstrate that the MRSA strain LAC can resist killing and grow in human synovial fluid from osteoarthritic knees. Furthermore, we show that the GraXRS regulatory system is required for the displayed synovial fluid resistance. We further demonstrate that a small-molecule inhibitor of GraR sensitizes LAC to synovial fluid, validating the Gra system as a therapeutic target for the treatment of PJIs in humans.

## INTRODUCTION

Osteoarthritis is the most prevalent joint disease in the United States and other developed countries, with osteoarthritis of the knee accounting for over 80% of the total disease burden (1, 2). Osteoarthritis is a degenerative disease that results in the breakdown of joint cartilage and underlying bone. Early symptoms include joint pain, stiffness, and swelling, but disease progression can significantly impact a patient’s quality of life. Fortunately, surgical intervention, including total joint replacement, is a clinically effective procedure that can be used to remedy joint disease (3). The number of joint replacement surgeries performed is increasing each year and is projected to reach 4 million annual cases in the United States by 2030 (4). Unfortunately, joint replacement procedures come with a risk of infection that is estimated to occur in 1 to 2% of cases (5). Moreover, periprosthetic joint infection (PJI) is a leading cause of implant failure requiring revision surgery (5). Improvements to surgical techniques, implant design, and operating environments have helped to reduce the prevalence of PJIs (6, 7), yet PJIs are still associated with immense physiological, psychological, and financial burdens (8).

Staphylococcus aureus is a Gram-positive facultative anaerobe that is found as a commensal species, colonizing the anterior nares of approximately 30% of the human population, but it is also an opportunistic pathogen (9). S. aureus is able to cause a wide array of diseases, ranging from mild skin and soft tissue infections to much more severe conditions, such as endocarditis, sepsis, and osteomyelitis (10). Furthermore, S. aureus is well known for its ability to cause PJIs and is the most frequently isolated organism from PJIs of the knee or hip (11). Additionally, there has been a frightening increase in the prevalence of methicillin-resistant strains, with nearly half of S. aureus isolates from PJIs in the United States being methicillin resistant (12). PJI by methicillin-resistant S. aureus (MRSA) poses a serious risk not only to the future success of the implanted joint but also to the patient’s life. Despite this, treatment options are severely limited, and therefore new therapeutics are desperately needed.

Synovial fluid is the neutral pH, viscous, non-Newtonian fluid within synovial joints, such as the knee, hip, or elbow, that functions to lubricate and cushion the joints during movement (13, 14). There is evidence to suggest that synovial fluid may also contribute to the innate immune defense against joint infection. Gruber et al. demonstrated that synovial fluid from patients’ knees had bactericidal activity against S. aureus, S. epidermidis, and S. pyogenes, which are all common causes of bone and joint infections (15). The mechanism by which the synovial fluid kills bacteria has not yet been elucidated, and a better understanding of the antibacterial nature of the synovial environment may facilitate the development of improved treatment strategies for PJIs. This study sought to characterize the antimicrobial activity of synovial fluid from osteoarthritic patients against S. aureus and to begin to elucidate genetic determinants that underlie resistance leading to S. aureus infections in this niche.

## RESULTS

### Human synovial fluid is antimicrobial toward S. aureus.

**FIG 1:**
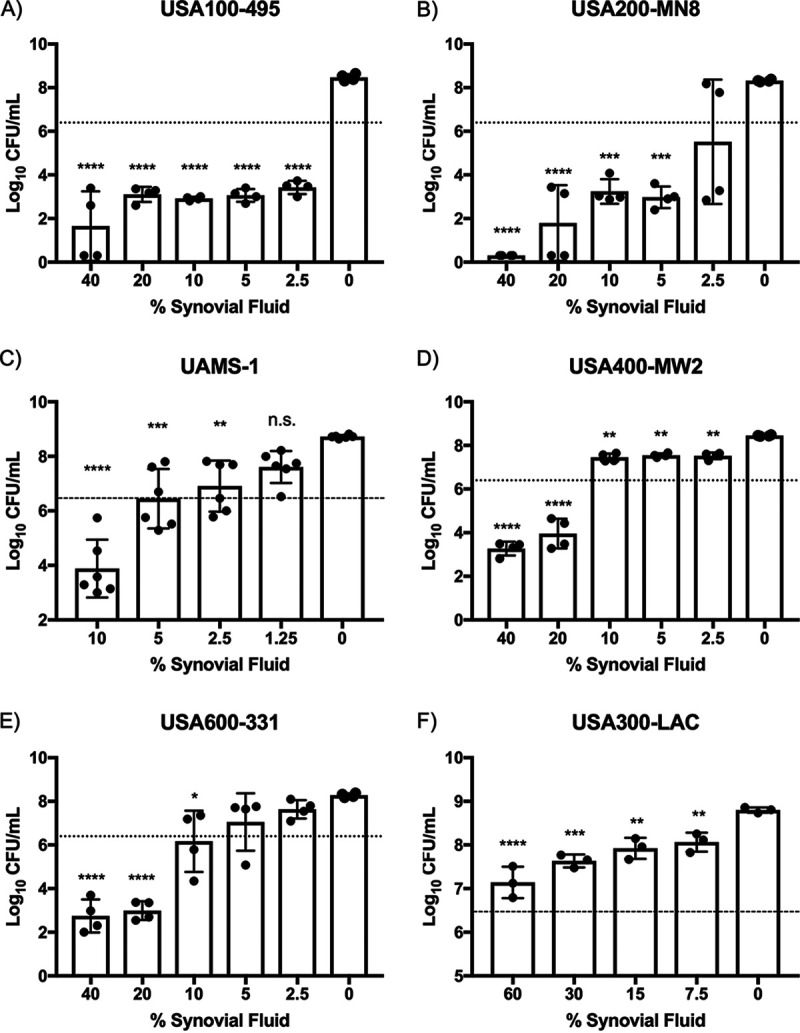
USA300-LAC is uniquely resistant to the antimicrobial activity of synovial fluid. (A to F) Abilities of S. aureus USA100-495 (A), USA200-MN8 (B), UAMS-1 (C), USA400-MW2 (D), USA600-331 (E), and USA300-LAC (F) to grow in various dilutions of synovial fluid taken from the knees of patients undergoing total knee replacements. Synovial fluid from an individual donor was diluted in RPMI 1640 medium and inoculated with ∼3 × 10^6^ CFU/ml, indicated by the horizontal dotted line. The number of CFU was determined by plating after a 24-h incubation at 37°C. Data shown are means ± SD, where each data point represents a biological replicate. All strains were exposed to synovial fluid from at least 3 of the same donors. Statistical significance was determined by a one-way analysis of variance (ANOVA) with Dunnett’s multiple-comparison posttest (*, *P* < 0.05; **, *P* < 0.01; ***, *P* < 0.001; ****, *P* < 0.0001). n.s., not significant.

**FIG 2:**
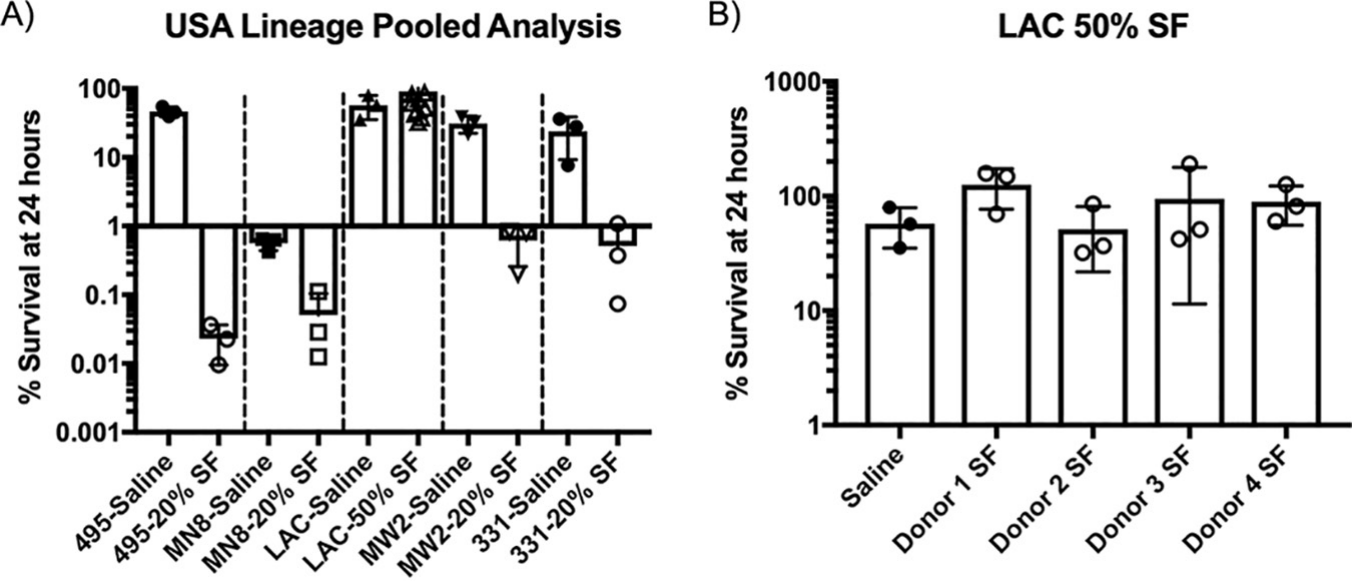
USA300-LAC is resistant to killing by synovial fluid in saline. (A) Abilities of S. aureus strains from various clonal lineages to survive in human synovial fluid (SF) that was pooled prior to dilution; (B) survival of LAC in synovial fluid from separate donors. Filled symbols indicate no-synovial-fluid controls, and hollow symbols indicate samples exposed to synovial fluid. Synovial fluid samples, both pooled and individual, were diluted in sterile saline and inoculated with ∼3 × 10^6^ CFU/ml. Percent survival was determined by drop plating to determine the number of CFU per milliliter remaining after 24 h. Data shown are means ± SD, where each data point represents a biological replicate.

To initially explore the effect of synovial fluid taken from osteoarthritic knees against S. aureus, growth assays were performed with a variety of S. aureus strains. We chose to use contemporary strains representing the most predominant clonal complexes (CCs) in human colonization and infection, including CC5 (USA100), CC30 (USA200), CC8 (USA300), CC1 (USA400), and CC45 (USA600), along with the osteomyelitis isolate UAMS-1. The bacteria were incubated at 37°C in RPMI 1640 with, or without, synovial fluid at various concentrations and then enumerated after 24 h. We found that the presence of synovial fluid significantly impacted the growth of all strains tested, although the levels of sensitivity varied greatly from strain to strain (Fig. 1). Strains 495 (USA100) and MN8 (USA200) were highly sensitive and displayed a reduction in bacterial viability over 24 h in as little as 2.5% synovial fluid (Fig. 1A and B). UAMS-1 was also significantly impacted, showing a reduction in growth in as little as 2.5% synovial fluid and significant killing in ≥10% synovial fluid (Fig. 1C). MW2 (USA400) and 331 (USA600) were somewhat more resistant than the previously discussed strains but were nonetheless sensitive to killing in ≥20% synovial fluid (Fig. 1D and E, respectively). Remarkably, in stark contrast to the other strains, strain LAC (USA300) retained the ability to replicate in synovial fluid concentrations as high as 60% (Fig. 1F). When incubated in synovial fluid pooled from multiple donors and diluted in saline rather than RPMI 1640 in order to prevent growth, these strains demonstrated similar patterns of sensitivity, with LAC being highly resistant to killing (Fig. 2A). To examine this resistance of LAC further, we then incubated LAC in saline containing synovial fluid separated by individual donor and observed that LAC was highly resistant to that taken from each donor (Fig. 2B). Taken together, the results indicate that there are antimicrobial factors in osteoarthritic synovial fluid that function to restrict and kill S. aureus independently of bacterial replication and that strain-to-strain variations in sensitivity exist, with the community-associated MRSA (CA-MRSA) strain LAC exhibiting a high level of resistance.

### Iron restriction contributes to the antimicrobial activity of synovial fluid.

**FIG 3:**
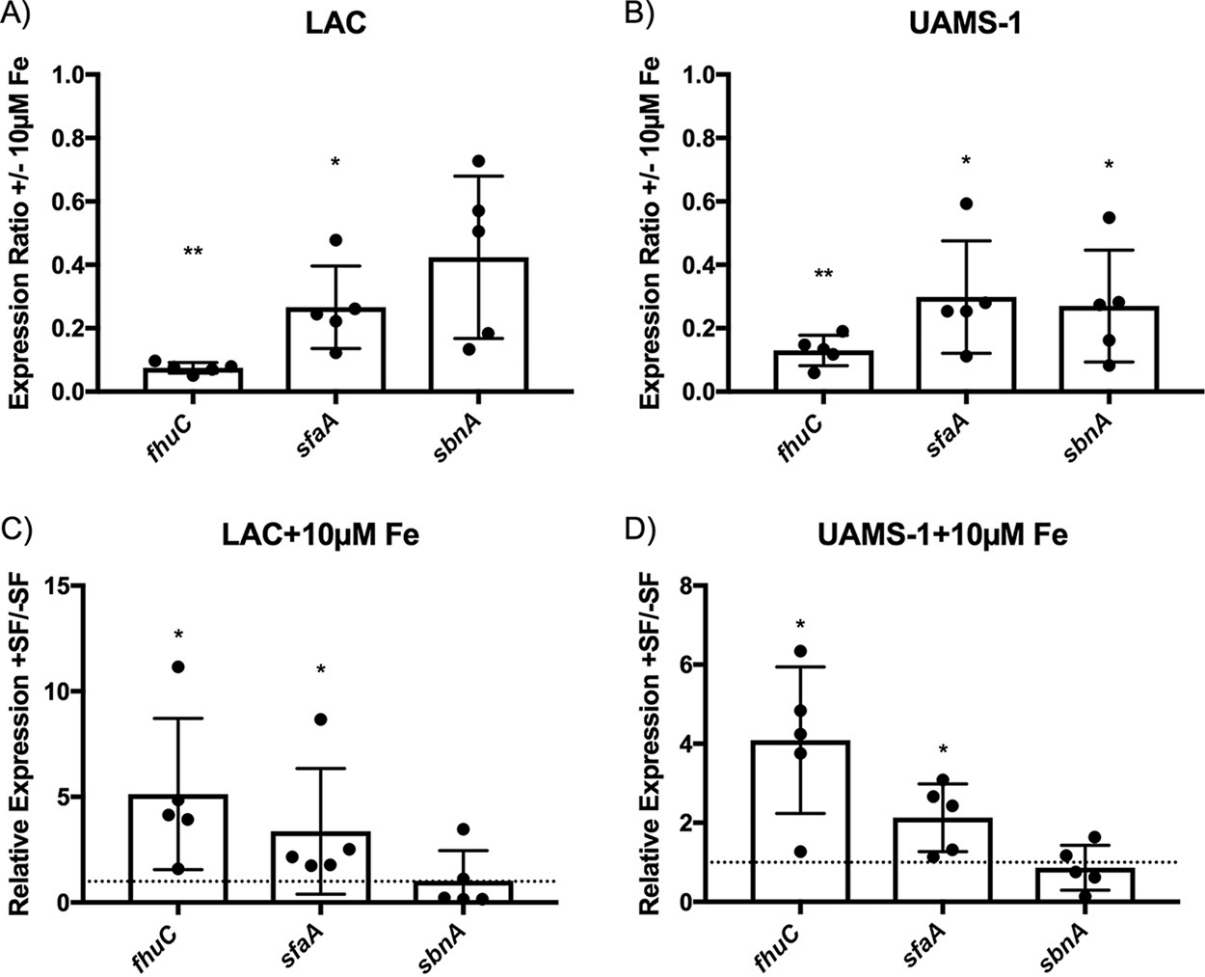
Synovial fluid enhances the expression of iron-regulated genes in S. aureus. Relative expression levels of the indicated genes in LAC (A) and UAMS-1 (B) grown in RPMI 1640 with and without the addition of 10 μM FeCl_3_ are shown. In addition, we show the relative levels of expression of the indicated genes in LAC (C) and UAMS-1 (D) grown in iron-replete RPMI 1640 with or without the addition of synovial fluid. The horizontal dashed line indicates a ratio of 1, indicating no difference between samples. Data presented are means ± SD, where each data point represents a biological replicate. Significance was determined using a paired-ratio *t* test on the ΔC_t_ values used to determine the expression ratios. *, *P* ≤ 0.05.

Having shown that synovial fluid is bactericidal against S. aureus, we next sought to determine which factors contribute to bacterial killing. To identify factors present in the patient samples employed in this study, a sodium dodecyl sulfate-polyacrylamide gel electrophoresis (SDS-PAGE) gel was run to determine the protein profiles of synovial fluids from different osteoarthritic donors. Various bands were excised and identified by matrix-associated laser desorption–ionization mass spectrometry (MALDI-MS), which confirmed the presence in synovial fluid of transferrin and several other proteins, which are listed in Table 1. Due to the role of transferrin in restricting bacterial access to iron within the human host (16–19), we next wanted to determine if synovial fluid is able to effectively sequester iron from S. aureus. To determine this, we examined the relative expression levels of several iron-regulated genes, with and without exposure to synovial fluid, in LAC and UAMS-1, which were chosen as representative resistant and sensitive strains and because of their relevance to bone and joint infections (20, 21). The bacteria were grown in RPMI 1640 containing 10 μM FeCl_3_ with, or without, supplementation with 5% synovial fluid for 2 h. Since RPMI 1640 is an iron-deficient growth medium, the iron was added in order to repress expression of iron-regulated genes in S. aureus, including *fhuC*, *sfaA*, and *sbnA*, which we chose to examine here as representative iron-regulated genes (22–24). We first confirmed the iron-regulated expression of these genes by showing that the addition of the iron to RMPI 1640 resulted in repressed expression of these genes in both LAC (Fig. 3A) and UAMS-1 (Fig. 3B) during growth. Next, we observed that during growth in iron-replete RPMI 1640, the addition of synovial fluid caused a significant increase in the expression of *fhuC* and *sfaA* in both LAC (Fig. 3C) and UAMS-1 (Fig. 3D). This result demonstrated that the synovial fluid imposed iron restriction upon the bacteria, likely through scavenging of the iron by transferrin present in the synovial fluid. Notably, although *sbnA* is an iron-regulated gene involved in the synthesis of staphyloferrin B, the relative expression of this gene did not change upon exposure to synovial fluid, in contrast to the expression of the other two aforementioned iron-regulated genes (Fig. 3A and B). We speculate this to be a consequence of the presence of trace amounts of heme in synovial fluid (25), as S. aureus uses heme as a signal to downregulate the expression of *sbn* genes (26).

**TABLE 1:** Proteins identified in osteoarthritic human synovial fluid

Protein	Molecular wt (kDa)	Function(s)	Protein score^*a*^
Immunoglobulin heavy chain	53	Large polypeptide subunit of antibodies that defines their isotype	116
Complement C3	144	Activates classical and alternative complement pathways for antibacterial defense	87
Factor H	143	Regulates activation of the complement system	198
Ceruloplasmin	122	Copper-carrying protein with copper-dependent ferroxidase activity	157
Transferrin	79	High-affinity Fe^3+^-binding protein	273
Albumin	71	Maintains osmotic pressure and acts as a plasma carrier protein	374
Apolipoprotein A-IV	43	Various functions in human lipid metabolism	154
Albumin	71	Maintains osmotic pressure and acts as a plasma carrier protein	169
Albumin	48	Maintains osmotic pressure and acts as a plasma carrier protein	146
Albumin	48	Maintains osmotic pressure and acts as a plasma carrier protein	131
Apolipoprotein A-I	29	Component of high-density lipoprotein in plasma; enables efflux of fat molecules from within cells for transport	292

a Protein scores are −10 times log(*P*), where *P* is the probability that the observed match is a random event. Protein scores greater than 68 are significant (*P* < 0.05).

**FIG 4:**
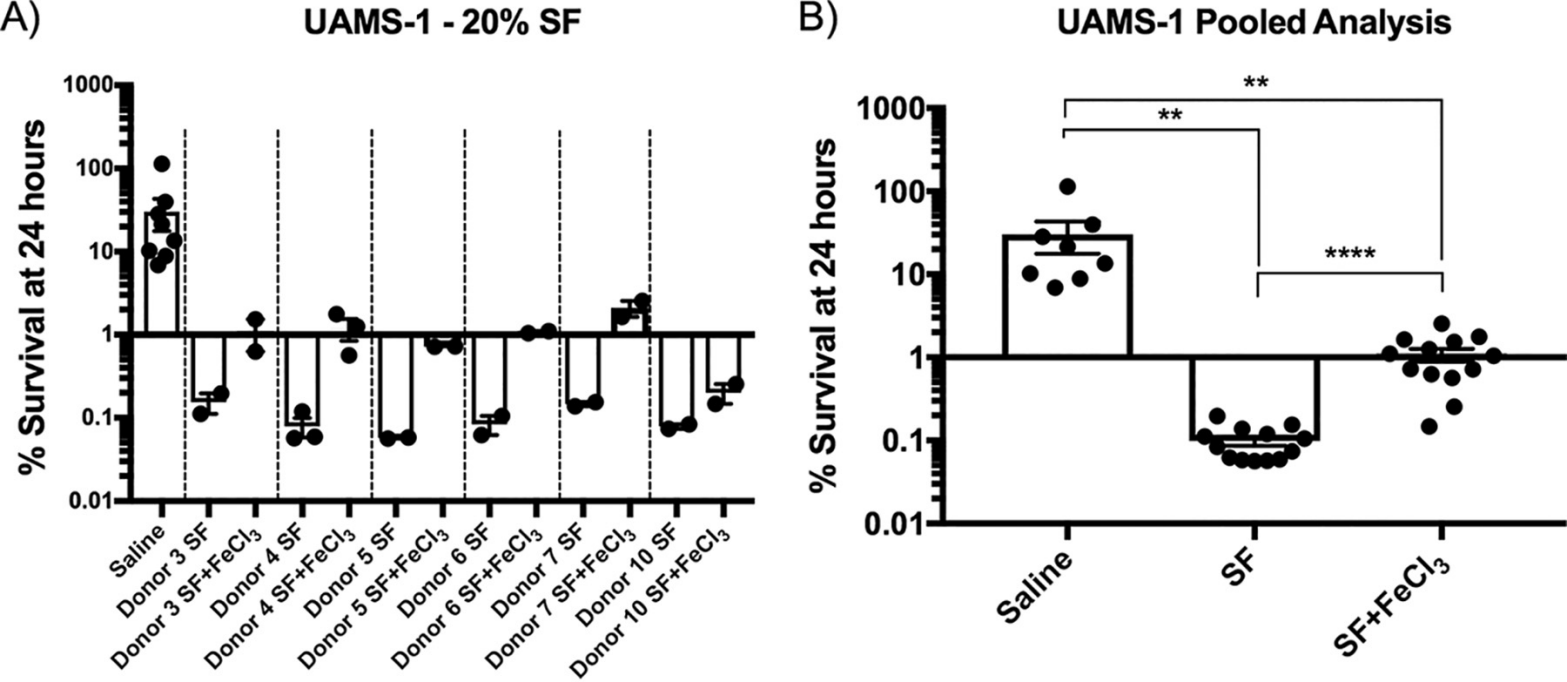
Iron restriction imposed by synovial fluid contributes to S. aureus killing. Bacterial viability assays were used to assess the ability of S. aureus UAMS-1 to survive in 20% human synovial fluid diluted in sterile saline solution with or without the addition of 25 μM FeCl_3_. The number of CFU for each sample was determined both at the time of inoculation and after 24 h at 37°C to determine the percent survival for each condition tested. (A) Results separated by each synovial fluid donor (dashed lines); (B) the same data in a combined statistical analysis. Data shown are means ± SD for 8 independent experiments. Statistical significance was determined by a one-way ANOVA with Tukey’s multiple-comparison posttest (*, *P* < 0.05; **, *P* < 0.01; ***, *P* < 0.001; ****, *P* < 0.0001).

**FIG 5:**
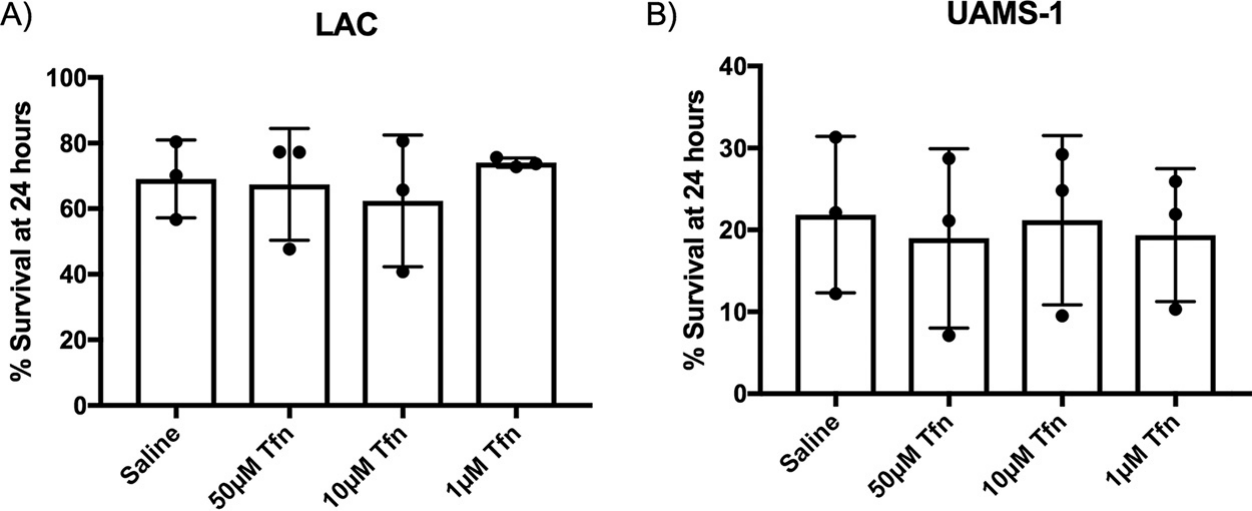
Transferrin alone is insufficient to cause bacterial death. Survival of S. aureus LAC (A) and UAMS-1 (B) following exposure to various concentrations of human transferrin (Tfn) in saline. Samples were plated at the time of inoculation and after 24 h at 37°C to determine the percent survival for each sample. Data shown are means ± SD from 3 independent experiments. All differences are nonsignificant as determined by an unpaired *t* test.

Having demonstrated that synovial fluid imposes iron restriction upon the bacteria, we next wanted to investigate whether or not iron limitation contributed to the bactericidal activity of synovial fluid. To this end, bacterial viability assays were performed in saline, which, again, is not permissive to bacterial replication, supplemented with synovial fluid with or without 25 μM FeCl_3_ as a source of free iron. Figure 4A shows the results of bacterial viability assays separated by individual synovial fluid donors, while Fig. 4B shows the same data but in a combined analysis. These data demonstrate that synovial fluid from a panel of donors is generally microbicidal toward UAMS-1 and that the addition of exogenous iron improved bacterial survival. This suggests that iron restriction in synovial fluid, presumably via scavenging of free iron by transferrin, leads to enhanced bacterial killing. Importantly, and as would be expected, transferrin alone is insufficient to cause bacterial death (Fig. 5). This indicates that the iron-restrictive environment of synovial fluid may function to sensitize the bacteria to killing by other factors.

### Disruption of *graXRS* signaling and/or *mprF* enhances synovial fluid killing.

**FIG 6:**
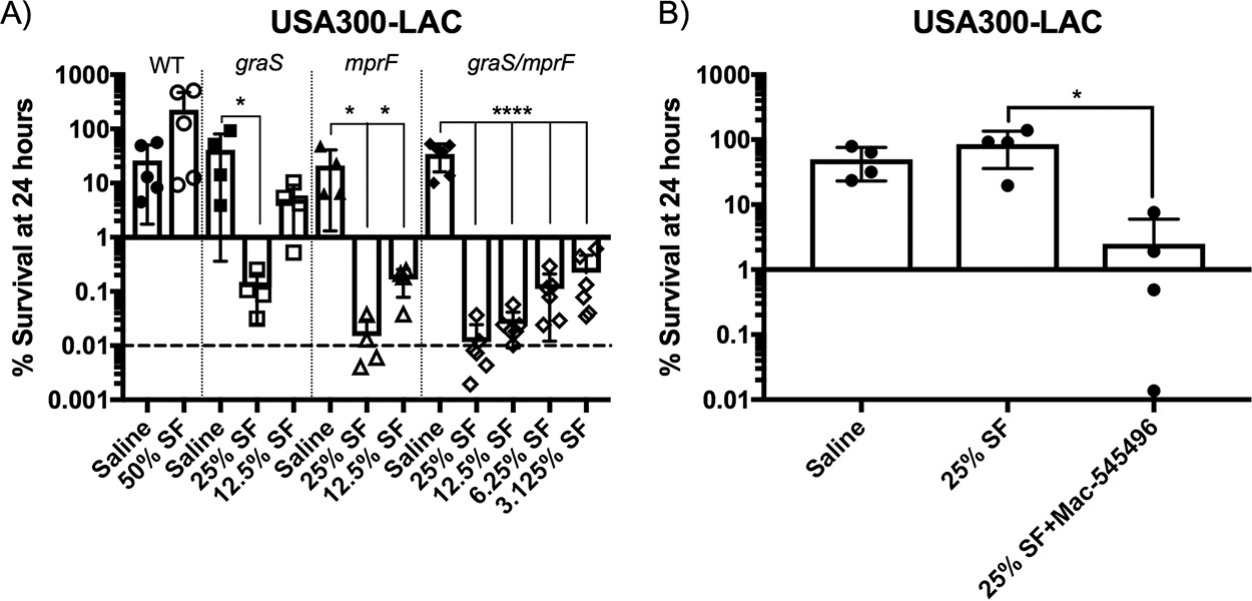
GraS regulation of *mprF* is required for resistance to killing by synovial fluid. (A) Ability of S. aureus LAC *graS*, *mprF*, and *graS mprF* mutants to survive in synovial fluid compared to that of the WT; (B) effect of the GraR inhibitor Mac-545496 (50 μM) on the survival of WT S. aureus LAC in synovial fluid. Viability assays were performed, and the percent survival was determined as described in the text. Data shown are means ± SD, where each data point represents a biological replicate. All strains in panel A were exposed to synovial fluid from at least 4 of the same donors. Statistical significance was determined using unpaired *t* tests (*, *P* < 0.05; ****, *P* < 0.0001). (A) Filled symbols indicate no-synovial-fluid controls, and hollow symbols indicate samples exposed to synovial fluid. The horizontal dashed line indicates the limit of accurate detection.

To gain insight into the mechanism of bacterial killing, we used the USA300 LAC strain because of its inherent high level of resistance to killing by synovial fluid and the availability of mutants defective for genes that have previously been shown to engender resistance to host innate immune factors. The cationic antimicrobial peptides (CAMPs) LL-37, human β defensin-2, and human β defensin-3 were previously identified in joint aspirates of patients with periprosthetic joint infections (27, 28). S. aureus senses and responds to low pH and CAMPs using the 5-component GraXRS-VraFG system (29–33). Thus, with this knowledge, we chose to next examine the effects of mutation of *graS* on sensitivity to synovial fluid. The *graS* mutant was therefore used in bacterial viability assays, and survival was compared to that of wild-type (WT) LAC in the presence or absence of synovial fluid. We observed that the LAC *graS* mutant was sensitive to killing in as little as 25% synovial fluid, whereas WT LAC was completely resistant in 50% synovial fluid, the highest concentration tested (Fig. 6A). Notably, GraXRS regulates the expression of the gene *mprF*, which catalyzes the lysinylation of phosphatidylglycerol in the cytoplasmic membrane to confer CAMP resistance (34, 35). Therefore, we also tested an LAC *mprF* mutant and found that it was further sensitized to synovial fluid killing. Indeed, a significant decrease in bacterial survival was observed in as little as 12.5% synovial fluid (Fig. 6A). When we combined mutations in *graS* and *mprF* into one strain, we observed that the LAC *graS mprF* mutant was hyper-sensitive, with significant bacterial killing in as little as 3.125% synovial fluid (Fig. 6A). We next demonstrated the efficacy of this regulatory system as a target for therapeutics in PJIs by employing a recently identified GraR inhibitor (36). Indeed, treatment with the GraR inhibitor sensitized WT LAC to killing in 25% synovial fluid (Fig. 6B); this level of sensitivity was similar to that observed for the *graS* mutant (Fig. 6A). These data demonstrate that S. aureus requires *mprF* for maximum resistance to killing by host factors, likely CAMPs, within synovial fluid; however, other GraXRS-regulated gene products play a role in resistance to the killing by synovial fluid.

### Relative gene expression levels in LAC and UAMS-1 correlate with resistance to synovial fluid.

**FIG 7:**
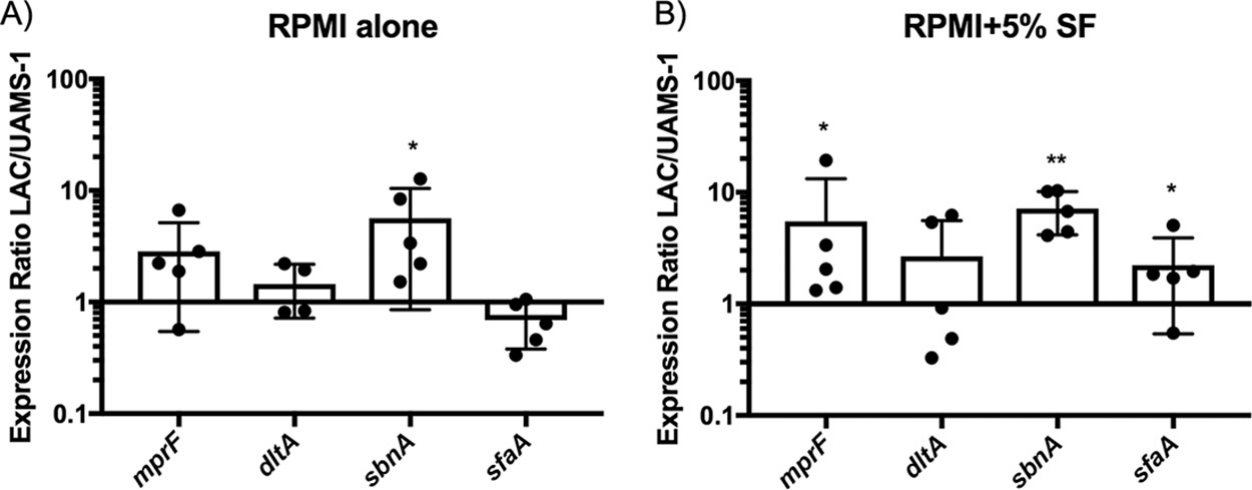
Relative gene expression for LAC compared to that of UAMS-1 in synovial fluid. (A and B) Relative expression of genes in LAC compared to UAMS-1 when cultured without (A) or with (B) 5% synovial fluid, as determined by qPCR. Bacteria were grown in RPMI 1640 to an OD_600_ of 0.5 and then supplemented with 5% synovial fluid from matching donor samples or left without synovial fluid and incubated for 2 h at 37°C. A number of cells equivalent to 1 ml of a culture at an OD_600_ of 3 were pelleted, and RNA was isolated for qPCR. The qPCR data for each strain were normalized to those for *rpoB* and then used to determine the relative expression of each gene in LAC compared to UAMS-1. Data shown are means ± SD, where each data point represents a biological replicate. Significance was determined using a paired-ratio *t* test on the ΔC_t_ values used to determine the expression ratios. *, *P* ≤ 0.05; **, *P* < 0.01.

Having demonstrated the roles of iron acquisition as well as *graS-*regulated *mprF* for survival in synovial fluid, we next measured the expression levels of *mprF*, *dltA*, *sbnA*, and *sfaA* for LAC (synovial fluid resistant) compared to those in UAMS-1 (synovial fluid sensitive) when cultured with and without synovial fluid. Strains were grown to an optical density at 600 nm (OD_600_) of 0.5 in RPMI 1640, which was then supplemented with 5% synovial fluid, or not, for 2 h, after which the expression levels of the aforementioned genes were measured using quantitative PCR (qPCR) and compared between strains. We found that LAC expressed higher levels of *sbnA* than UAMS-1, with and without exposure to synovial fluid, and that synovial fluid led to significantly increased expression of *mprF* and *sfaA* by LAC relative to that in UAMS-1 (Fig. 7). With inherently higher levels of expression of these genes, LAC may be better suited than UAMS-1 to respond to antimicrobial effector molecules in synovial fluid. However, as previously stated, other unidentified GraXRS-regulated factors contribute to the survival of LAC in synovial fluid. Thus, we also investigated the expression levels of *dltA*, another GraXRS-regulated resistance gene (30), although no difference in expression was observed between strains with or without synovial fluid (Fig. 7). This is an indication that *dltA* expression levels may not be responsible for the difference in observed resistance between strains. Altogether, these analyses demonstrated that iron acquisition and GraXRS-regulated resistance genes play a key role in the survival of S. aureus within human synovial fluid and suggest that increased expression of key genes by LAC contribute to its high level of resistance to killing by synovial fluid compared to that of other strains.

## DISCUSSION

The number of arthroplasty procedures performed increases each year, and as a result, a subsequent increase in revision arthroplasty procedures is expected as well (37). PJI is a leading cause of revision surgery, presents a significant challenge to the success of a joint replacement, and often results in significant patient morbidity. Despite the ability of S. aureus to cause PJI, there has been relatively little research regarding the effects of synovial fluid on this bacterium. Our data demonstrate that synovial fluid from the knees of osteoarthritic patients is iron restrictive and bactericidal against S. aureus. Indeed, we have shown that iron restriction potentiates the bactericidal action of synovial fluid and that S. aureus mutants lacking the ability to express *mprF* and/or *graS* have increased sensitivity to synovial fluid-dependent killing.

It has become evident that iron is actively sequestered in synovial fluid from osteoarthritic patients due to the activity of host iron-binding proteins, such as transferrin. In a previous study, it was found that transferrin was abundant in the synovial fluid from osteoarthritic patients compared to that from patients with rheumatoid arthritis, which showed lower levels of transferrin and increased free iron (17). Presumably, the presence of transferrin helps curtail bacterial growth within the infected joint, as iron is a critically important nutrient that is required by virtually all bacteria (16, 38, 39). Here, we confirmed the presence of transferrin in the synovial fluid of osteoarthritic patients and demonstrate the ability of synovial fluid to restrict bacterial access to iron, leading to increased expression of siderophore synthesis and uptake genes by S. aureus (Fig. 3). A similar result was observed by Xu et al., who demonstrated increased expression of siderophore biosynthetic loci when S. aureus was grown in joint fluid aspirate (40). We found that the survival of WT S. aureus was increased by adding free iron to the synovial fluid (Fig. 4), consistent with the notion that iron restriction inhibits bacterial growth. This suggests that in cases where additional iron is present in synovial fluid, S. aureus can utilize siderophores in order to access the transferrin-bound iron, allowing for improved survival and/or replication.

We found that S. aureus relies on the GraXRS-VraFG five-component regulatory system, in addition to siderophores, to survive in synovial fluid. Moreover, we found that deletion of the GraXRS-regulated *mprF* gene further enhanced sensitivity to synovial fluid, suggesting the presence of CAMPs in the fluid (30, 35). Of note, previous reports have identified various CAMPs in synovial fluid, including the cathelicidin LL-37, β defensins, and dermcidin, although the expression levels of the peptides vary depending on the disease type (rheumatoid/osteoarthritis) and infection status (25, 27, 28). Our data on the differences in sensitivity between *graS* and *mprF* mutants are consistent with previous reports where *graS* and *mprF* mutants in a murine infection model were attenuated compared to WT bacteria, but the level of attenuation was greater for *mprF*-deficient bacteria (29). Conceivably, the differences in sensitivity between the *graS* and *mprF* mutants are due to the contribution of the BraRS system in regulating the expression of *mprF*, which would allow for some expression of *mprF* in a *graS* mutant (41). Furthermore, a mutant lacking both *graS* and *mprF* exhibited an even greater level of sensitivity than either single mutant (Fig. 6A). This indicates that both *mprF* and other unknown GraXRS-regulated genes, potentially *dltABCD*, which encode enzymes essential for incorporation of d-alanine into cell wall polymers, are required for resistance to killing by synovial fluid. Additionally, we have shown that LAC expresses significantly larger amounts of *mprF* than UAMS-1 when challenged with synovial fluid (Fig. 7). This result is similar to the results of another study that observed that a low-virulence isolate of S. aureus from an implant-associated bone infection harbored frameshift mutations in multiple genes, including *graS* (42). It is tantalizing to speculate that the strain-to-strain differences in survival observed in this study are due to sequence differences in GraXRS-VraFG, which has approximately 90% sequence homology between LAC and UAMS-1, resulting in altered levels of function or expression of the various genes required for survival. Further detailed work is needed to identify the genetic basis of strain-to-strain variations in sensitivity to synovial fluid.

This work highlights the critical importance of the GraXRS regulatory system and the Gra-regulated *mprF* gene as a resistance system against innate immune effectors. Previous work has shown that GraS is able to sense acidic pH, such as that of human skin, or the phagolysosome of macrophages and respond through GraR-mediated upregulation of resistance genes, such as *mprF*, allowing for bacterial survival (29, 31, 43). However, as stated previously, it is known that synovial fluid from osteoarthritis patients has a neutral pH of approximately 7.35 (14), so GraS must sense some factor or condition, possibly CAMPs, in the synovial fluid other than the pH in order to respond through GraR-mediated gene expression. It is also worth noting that GraXRS is an intramembrane Bce-like sensing system, where the sensor kinase GraS requires the concerted action of the associated ABC transporter VraFG in order to sense CAMPs and transfer the received signal to their cognate response regulator, GraR (33, 44–46). Due to the reliance of GraS on VraFG, one might surmise that VraFG is also likely to play a key role in the survival of S. aureus in human synovial fluid, although more work is needed to characterize this relationship.

Due to the importance of the GraXRS system for S. aureus to survive in various niches within the host, it is likely that therapeutics targeting this system, or the genes regulated by this system, will be effective in treating various infections caused by S. aureus. Notably, a recently identified inhibitor of GraR was found not only to attenuate virulence in multiple infection models but also reversed β-lactam resistance in LAC (36). Here, we have shown that the GraR inhibitor is also able to effectively sensitize S. aureus LAC to killing by human synovial fluid (Fig. 6B). Thus, our data support the use of the GraR inhibitor as an adjuvant for the treatment for PJIs; however, more work is needed to determine the safety and efficacy of the inhibitor in PJI models.

Ultimately, this work has demonstrated that synovial fluid from osteoarthritic patients is iron restrictive and bactericidal to S. aureus, which requires the GraXRS-regulated expression of *mprF* and other as-yet-undefined genes in order to resist killing by the fluid. Thus, we propose GraXRS and/or MprF as targets for the continued development of novel therapeutics and treatment of PJIs caused by S. aureus.

## MATERIALS AND METHODS

### Synovial fluid isolation/preparation.

Synovial fluid samples were obtained from patients undergoing elective primary total knee replacement for treatment of symptomatic end-stage osteoarthritis. All samples were collected under sterile conditions by the same surgeon in the operating room at the University Hospital of the University of Western Ontario. As per the standard of care, to minimize the potential for contamination with blood, a tourniquet was applied prior to making the initial skin incision. The extensor mechanism and joint capsule were exposed, after which an 18-gauge needle in a 20-cm^3^ syringe was inserted into the joint anteromedially and the fluid was withdrawn from the knee.

The fluid was then spun in a centrifuge for 10 min at 4,700 × *g* to remove any possible red blood cell and lymphocyte contamination. An aliquot of the cell-free fluid was tested to ensure sterility, while the rest was transferred to sterile cryotubes and stored at −80°C, separated by donor, but no identifiable data were retained with samples. Patients with severe medical comorbidities, a diagnosis of inflammatory arthropathy (i.e., rheumatoid arthritis, gout), or a diagnosis of fibromyalgia were excluded. All samples were tested individually and were not pooled unless otherwise stated. Patients were monitored at their standard follow-up appointments with the surgeon for adverse events or complications according to the standard of care at the University Hospital. There was no follow-up monitoring for this study.

### Bacterial strains and culture conditions.

S. aureus strains used in this study are listed in Table 2. Bacteria were routinely cultured at 37°C in liquid tryptic soy broth (TSB) (Difco) with shaking overnight at 200 rpm or on solid TSB agar (1.5%, wt/vol) (TSA) plates. Strains bearing transposon insertions were cultured in the presence of 3 μg/ml erythromycin.

**TABLE 2:** Bacterial strains and qPCR primers used in this study

Strain or primer	Description or sequence	Source or primer amplicon size (bp)
Strains		
UAMS-1	Osteomyelitis isolate; methicillin-susceptible S. aureus	20
495	USA100 (CC5)	J. K. McCormick
MN8	USA200 (CC30); methicillin-susceptible S. aureus	48
LAC	USA300-LAC (CC8) CA-MRSA; cured of antibiotic resistance plasmid	49
MW2	USA400 (CC1) MRSA	50
331	USA600 (CC45)	51
LAC *graS*	LAC *graS*::ϕNΣ Ery^r^	52
LAC *mprF*	LAC *mprF*::ϕNΣ Ery^r^	52
LAC *graS mprF*	LAC Δ*graS mprF*::ϕNΣ Ery^r^	This study

Primers		
*rpoB-*F	AGAGAAAGACGGCACTGAAAACAC	156
*rpoB-*R	ATAACGACCCACGCTTGCTAAG	
*mprF-*F	CGTGTCTTTGAACATTTCAACGGTC	137
*mprF-*R	ACTTTAGAAAGTGATTCCCAAAGCGA	
*dltA-*F	GCAACACCGATTAACATTTGGGTATC	177
*dltA-*R	TGCTCTGTGAGGTAGAATTTCACC	
*fhuC-*F	ATCATTGGTCCTAACGGCTGCG	174
*fhuC-*R	GCCATCTGCTACTTCAGGTGATTGAG	
*sfaA-*F	GCACTTAAAATCCCTCTTAATGTCGC	92
*sfaA-*R	TACTAAGTGTGTGGGCAGGGG	
*sbnA-*F	CATGGTGCAGGCACAGAGATTGTT	178
*sbnA-*R	ATACCCATAATGCTACCTGTCGTGC	

### S. aureus growth in synovial fluid.

Aliquots of frozen human synovial fluid samples from individual donors were thawed and diluted in the chemically defined medium RPMI 1640 immediately prior to use for growth assays with S. aureus. Overnight cultures of the bacteria were grown in 5-ml cultures of TSB and normalized to an OD_600_ of 1.0 in sterile saline. Tubes containing a 2-fold dilution series of synovial fluid in RPMI 1640 were inoculated with 10 μl of the bacterial suspension at an OD_600_ of 1.0 to start each tube at an initial OD_600_ of 0.01, equivalent to ∼2 × 10^6^ to 4 × 10^6^ CFU/ml. The samples were incubated at 37°C with shaking at 200 rpm for 24 h, after which the samples were serially diluted and 10 μl was drop plated on TSA plates. After overnight incubation, the colonies were counted to determine the final number of CFU per milliliter for each sample.

### Bacterial viability assay.

Viability assays were performed to determine the ability of various strains and mutants of S. aureus bacteria to survive in synovial fluid. As before, aliquots of synovial fluid were thawed immediately prior to use and were diluted in saline in order to assess bacterial survival/death in the absence of replication. FeCl_3_, transferrin (Sigma-Aldrich), or the GraR inhibitor Mac-545496 (Cedarlane) were added when needed at the concentrations indicated in the figure legends. Bacteria were grown overnight in tubes containing 5 ml of TSB and with 1 μM Mac-545496 when needed and normalized to an OD_600_ of 1.0 prior to inoculation at an initial OD_600_ equivalent to 0.01 in growth medium containing synovial fluid. All samples were plated immediately after inoculation to determine the starting number of bacteria, expressed as CFU per milliliter. Samples were again plated after a 24-h incubation at 37°C to determine the number of bacteria remaining.

### Identification of proteins in synovial fluid.

Synovial fluid samples from random patients were thawed and diluted to an initial concentration of 20% (vol/vol) in saline. Then, 10 μl of the diluted fluid from different donors was mixed 1:1 with Laemmli buffer and boiled for 10 min. After cooling, 7 μl of each sample was loaded and run on a 12% sodium dodecyl sulfate-polyacrylamide gel in Tris-glycine buffer at 150 V and 500 mA for 90 min. Upon completion, the gel was stained with InstantBlue, and an automated spot picker at the London Regional Proteomics Centre was used to excise individual protein bands from the gel. The samples were digested with trypsin, and MALDI-MS was performed on the digested samples.

### RNA isolation and RT-qPCR.

Matching cultures were grown in RPMI 1640, with and without 10 μM FeCl_3_, to an OD_600_ of 0.5 and then either supplemented with 5% (vol/vol) synovial fluid or kept as no-synovial-fluid controls and incubated for 2 h at 37°C with shaking. A volume of each culture containing the equivalent number of cells as a 1-ml culture at an OD_600_ of 3 was mixed with a 2× volume of RNAprotect (Qiagen) and incubated at room temperature for 5 min before being pelleted and frozen. RNA was isolated using an RNeasy minikit (Qiagen), and 500 ng of RNA was reverse transcribed using a one-step reverse transcription (RT)-PCR kit (Bio-Rad) according to manufacturer’s instructions. cDNA was PCR amplified using iTaq SYBR green (Bio-Rad), and data were normalized to data for the reference gene, *rpoB.* Primers used are listed in Table 2. Expression ratios were calculated using the previously established ΔΔC_t_ method (where C_t_ is the cycle threshold) (47), and statistical significance was determined using ratio-paired *t* tests on the ΔC_t_ values used to generate the expression ratios.

### Statistical analysis.

Data are presented as means ± standard deviations (SD). All statistical analyses and graph production were done through GraphPad Prism (GraphPad Software, La Jolla, CA).

### Ethics statement.

Prior to patient enrollment for the synovial fluid collection, this research was approved by the Health Sciences Research Ethics Board of the University of Western Ontario and Lawson Health Research Institute. An informed consent was obtained from each patient prior to the procedure.
